# Evaluation of a Machine Learning-Based Dysphagia Prediction Tool in Clinical Routine: A Prospective Observational Cohort Study

**DOI:** 10.1007/s00455-022-10548-9

**Published:** 2023-01-10

**Authors:** Stefanie Jauk, Diether Kramer, Sai Pavan Kumar Veeranki, Angelika Siml-Fraissler, Angelika Lenz-Waldbauer, Ewald Tax, Werner Leodolter, Markus Gugatschka

**Affiliations:** 1 Information and Process Management, Steiermärkische Krankenanstaltengesellschaft M.B.H. (KAGes), Billrothgasse 18a, 8010 Graz, Austria; 2 Steiermärkische Krankenanstaltengesellschaft M.B.H. (KAGes), LKH Weststeiermark, Conrad-von-Hötzendorf-Straße 31, 8570 Voitsberg, Austria; 3 Division of Phoniatrics, Department of Otorhinolaryngology, Medical University of Graz, Auenbruggerplatz 26, 8036 Graz, Austria

**Keywords:** Dysphagia, Machine learning, Clinical decision support, Risk assessment, Predictive modeling, Electronic health records

## Abstract

Based on a large number of pre-existing documented electronic health records (EHR), we developed a machine learning (ML) algorithm for detection of dysphagia and aspiration pneumonia. The aim of our study was to prospectively apply this algorithm in two large patient cohorts. The tool was integrated in the hospital information system of a secondary care hospital in Austria. Based on existing data such as diagnoses, laboratory, and medication, dysphagia risk was predicted automatically, and patients were stratified into three risk groups. Patients’ risk groups and risk factors were visualized in a web application. Prospective predictions of 1270 admissions to geriatric or internal medicine departments were compared with the occurrence of dysphagia or aspiration pneumonia of routinely documented events. The discriminative performance for internal medicine patients (*n* = 885) was excellent with an AUROC of 0.841, a sensitivity of 74.2%, and a specificity of 84.1%. For the smaller geriatric cohort (*n* = 221), the AUROC was 0.758, sensitivity 44.4%, and specificity 93.0%. For both cohorts, calibration plots showed a slight overestimation of the risk. This is the first study to evaluate the performance of a ML-based prediction tool for dysphagia in a prospective clinical setting. Future studies should validate the predictions on data of systematic dysphagia screening by specialists and evaluate user satisfaction and acceptance. The ML-based dysphagia prediction tool achieved an excellent performance in the internal medicine cohort. More data are needed to determine the performance in geriatric patients.

**Supplementary Information:**

The online version contains supplementary material available at 10.1007/s00455-022-10548-9.

## Introduction

In everyday clinical practice, clinicians need to make various decisions regarding diagnosis or treatment of patients. An essential base for clinical decision-making is the estimation of a patient’s risks for certain clinical events. Clinical prediction models aim to support healthcare professionals when dealing with such risk evaluation. Although the majority of these models were developed using large cohort studies or established clinical guidelines [1], various attempts have been made over the last years to develop models based on machine learning (ML) and artificial intelligence (AI) [2].

A major advantage when using ML-based models is their ability to analyze patterns in big amounts of data, overcoming limitations of human cognition [3]. For instance, data stored in electronic health record (EHR) systems can be retrieved to train clinical prediction models. The use of routinely documented patient data and ML algorithms further personalizes patient care [4] and allows for fast and automatic risk predictions without additional data entry in information systems. However, although many ML models achieved good performance in test data, few of them have been implemented in clinical settings [2] and there has been little prospective validation [3].

Various clinical prediction models and derived risk scores have been developed for the prediction of aspiration pneumonia over the past years [5–8]. The use of such models for early screening can be effective in preventing aspiration pneumonia in dysphagia patients, e.g., following an ischemic insult [5]. The scoring model SSG-OD developed by Zhou et al. [8] achieved a sensitivity of 68.5% and a specificity of 89%. The AUROC of the RODICx score [9] on a test data set was 0.75. The PreDyScore for predicting persistent dysphagia developed by Gandolfo et al. [10] had a sensitivity of 67% and a specificity of 95.7% (AUROC 0.79) in the test data set.

However, most models to date are limited in two aspects. First, they focus on very specific risk populations such as Parkinson patients [9], patients after anterior cervical spine surgery [11], or cardiac surgery [6, 7]. Second, their conduction is time consuming and requires personal resources.

An ideal dysphagia screening should be quick, non-invasive, and should allow a broad application across different populations of hospitalized patients. Few publications reported more generalizable prediction models, and for many models, additional clinical assessments are needed [12, 13]. These limitations highlight the need for different approaches, e.g., the combination of ML and routinely documented EHR data for risk prediction.

The aim of this study was to evaluate a ML-based tool predicting dysphagia and aspiration pneumonia based on large amounts of existing data and new data obtained by prospectively included patients. For the evaluation, the tool was implemented in the hospital information system (HIS) of a secondary care hospital within a pilot study. The evaluation focused on the performance of the model in the dynamic clinical setting.

## Methods

### The Risk Prediction Algorithm

In 2019, we developed a ML-based model predicting dysphagia or aspiration pneumonia in hospitalized patients [14]. The predicted outcome was defined as a coded diagnosis for dysphagia (R13) or aspiration pneumonia (J59) during the hospital stay according to the International Classification of Diseases, tenth revision, German modification (ICD-10-GM).

In order to achieve the best performance, we trained various ML techniques such as random forests, artificial neural nets, and gradient boosting machines on routinely collected EHR data of public hospitals in Styria, a county of Austria. The data are hosted by KAGes (Steiermärkische Krankenanstaltengesellschaft m.b.H.), the regional healthcare provider in Styria, which uses an EHR system with longitudinal EHR data of more than 2 million patients from the region.

Routinely stored EHR data of 33,784 patients from 2011 until 2019 were retrieved from the EHR system of KAGes. Data included ICD-10-GM coded diagnoses, procedures, laboratory values, nursing data, medication, demographic, and administrative data. After feature selection, the data set resulted in 886 features for prediction. The most prevalent diagnoses features are presented in Table 1. Features were divided in seven groups (see Table 2), a comprehensive display of features employed can be found in the Supplement section. On the unseen test data, a random forest model achieved an excellent performance with an area under the receiver operating-characteristic curve (AUROC) of 0.94, and a sensitivity and specificity of 88% [14].

**Table 1:** Descriptive statistics of analyzed hospital admissions including dysphagia relevant diagnoses in the electronic health records

	Internal medicine ward	Geriatric ward	Total
N (admissions)	1048	222	1270
Age, years^a^	74.5 (63.00–82.00)	79.0 (71.00–85.75)	76.0 (64–83)
Length of stay, days^a^	6.1 (3.16–9.85)	20.0 (15.06–27.03)	7.0 (3.87–13.95)

	*N*	%	*n*	%	*n*	%
Sex, *n*						
M	562	53.6	75	33.8	637	50.2
F	486	46.4	147	66.2	633	49.8

	ICD-10-GM codes	*n*	%	*n*	%	*n*	%
Previously coded diagnoses in patient history							
Dysphagia	R13	22	2.1	2	0.9	24	1.9
Pneumonitis due to solids and liquids	J69.0, J69.9	15	1.4	2	0.9	17	1.3
Cerebral infarction, stroke	I63, I64	95	9.1	20	9.0	115	9.1
Dementia, Alzheimer	F00, F01, F02, F03, G30	62	5.9	15	6.8	77	6.1
Parkinson disease	G20, G21, G22	35	3.3	10	4.5	45	3.5
Diseases of vocal cords and larynx	J38	7	0.7	2	0.9	9	0.7
Diseases of esophagus	K20, K21, K22	135	12.9	24	10.8	159	12.5
Malignant neoplasms of lip, oral cavity and pharynx	C00-C14, C32, C33	7	0.7	1	0.5	8	0.6

Values are presented as absolute frequencies (column percentages)

^a^Median (Q1–Q3)

**Table 2:** Feature set for the prediction of dysphagia from EHR data with examples for the seven feature groups [14]

Data type	Description	*n*
Demographic data	Age, gender	28
Diagnosis codes	ICD-10 codes, groups of ICD-10 Codes	286
Procedures codes	Examinations and procedures: MRI, CT	103
Laboratory data	Thrombocytes, creatinine	190
Nursing protocols	Body mass index, movement disorders	92
Administrative data, indices	Charlson Comorbidity Index, number of hospital stays	25
Medication	Medication associated with dysphagia	162

The developed model was trained for predicting dysphagia or aspiration pneumonia on the second evening after admission (Model C). However, for a successful deployment in clinical routine, risk prediction should be available as early as possible. Therefore, we trained two additional models for predicting the risk at time of admission (Model A) and at the evening of admission (Model B). As for Model C, the random forest algorithm outperformed other algorithms [14], we retrained random forests for the additional prediction times accordingly. Using the same algorithms made it easier to use the same data model, which further simplified implementation. The advantage of prediction some hours after admission is that recent laboratory values and nursing assessments can be included in the prediction. Table A1 and Fig. A1 in Supplement A present details on the performance of the three models integrated in the tool.

### Integration and Visualization in the Hospital Information System

The integration of the prediction in the HIS and the visualization in the dysphagia risk prediction tool was based on our previous implementation of a tool predicting delirium in hospitalized patients [15]. For every patient admitted to a hospital department, an HL7 message was sent from the HIS to a local hospital server, and patient data needed for prediction were retrieved using http-requests. A prediction algorithm running on the server predicted dysphagia for each patient at admission time with Model A and recalculated the risk at the evening of admission and the evening of the following day using Model B and C, respectively. The recalculation included the most recent laboratory results and nursing assessment data. At each prediction time, the random forest models predicted the risk of dysphagia for each patient. All risk predictions and features values were stored in a data warehouse.

Based on the predicted dysphagia risk, each patient was stratified to one out of three risk groups. Thresholds determining the risk groups were chosen in concordance with clinicians; for the pilot study, 5% of patients with the highest dysphagia risk were stratified to the *very high risk* group, the following 10% to the *high risk* group, and the following 85% to the *low risk* group.

**Fig. 1:**
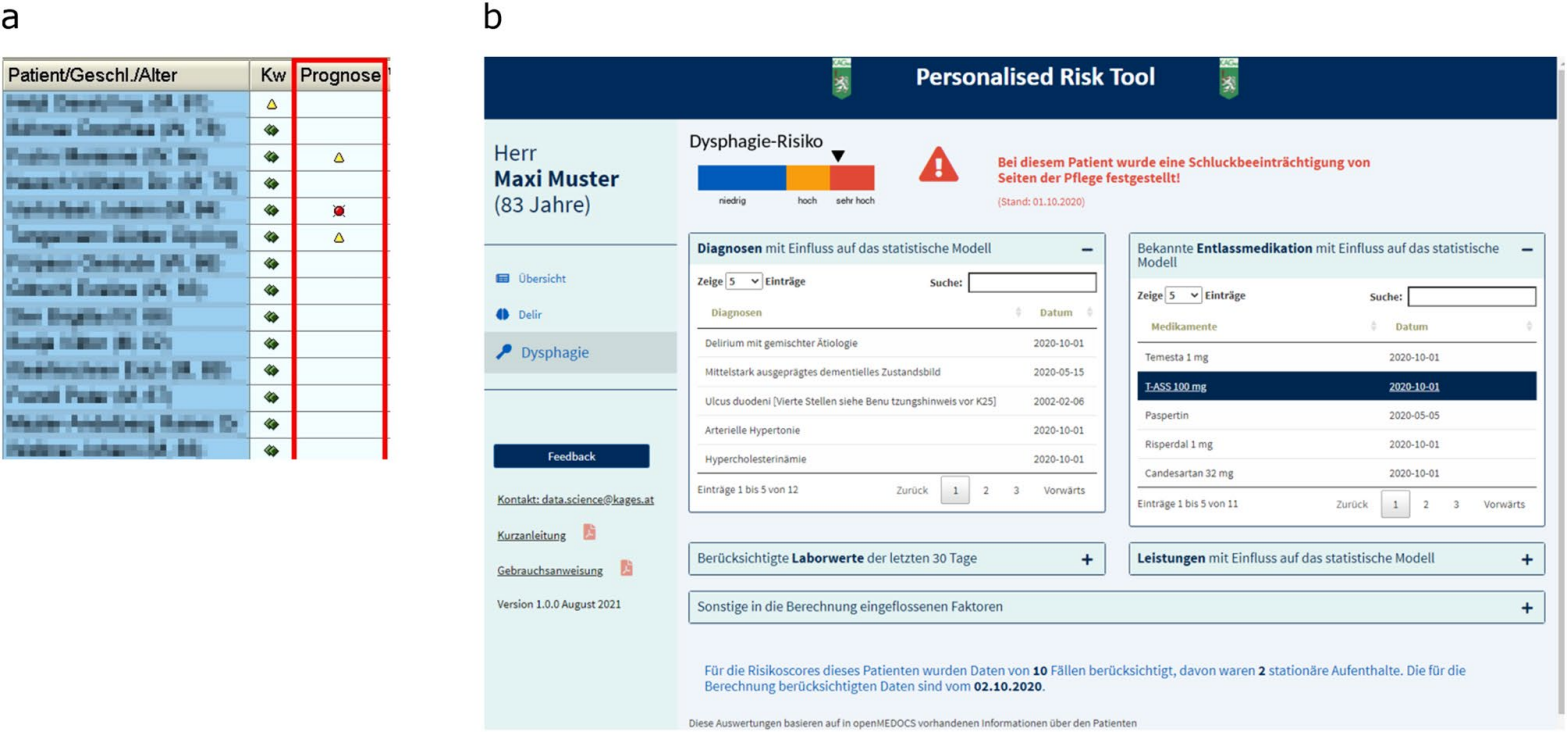
Visualization of the machine learning-based dysphagia prediction in the hospital information system (**a**) [23] and in a web application (**b**). This screenshot is fictional and not referring to a real patient

A patient’s risk was displayed to healthcare professionals using two presentation methods. First, a column named *Prognose* (German for prediction) was integrated in the user interface of the HIS. A red icon symbolized patients at *very high risk* (95th to 100th percentile) and a yellow icon those at *high risk* (85th to 94th percentile), shown in Fig. 1a. In order to avoid an information overflow at the user interface, no symbol was shown for *low risk* patients.

Second, a click on the icon or empty cell opened up a web application developed in R shiny [16], which provided patient-specific information relevant for prediction. The aim of the application was to facilitate decision-making of healthcare professionals and increase the usability of the ML-based risk prediction (see Fig. 1b).

### Study Design

In October 2020, the dysphagia prediction tool was first implemented in a KAGes hospital in Styria. The hospital treats different patient cohorts at two locations: At the first location, an internal medicine ward was included in the study, and at the second location, a geriatric ward including a remobilisation facility was included. After several training sessions, the tool was integrated in the user interface of physicians and nurses of both departments.

A prospective observational cohort study design was used to answer the research question. To identify patients with an occurrence of dysphagia or aspiration pneumonia as precise as possible, various sources of EHR data within the EHR system were used to identify a relevant clinical outcome. First, patients with an ICD-10-GM coded diagnosis for *dysphagia* (R13) or *pneumonitis due to solids and liquids* (J69.0/J69.9) were labeled as dysphagia patients. Second, patient’s discharge summaries and clinical notes of speech-language pathologists were screened for indication of dysphagia or aspiration pneumonia using search terms defined by clinicians (see Table A2 in Supplement A). Documents with a positive screening result were manually checked, and patients with clear evidence of dysphagia or aspiration pneumonia were included as dysphagia patients. Third, patients with documented procedures related to dysphagia (such as dysphagia therapy or dysphagia examination) were labeled as dysphagia patients.

All prospective predictions of the algorithm from 16th of October 2020 until 31st of October 2021 were included in the analysis. Patients younger than 18 years were excluded from the prediction. The analysis was performed at the level of hospitalization. For some hospitalizations, various risk predictions were available over their hospital stay due to internal transfers between the wards. Therefore, only the latest risk prediction within the first 48 h after admission or transfer was used for evaluation.

The study received approval from the Ethics Committee of the Medical University of Graz (30-146 ex 17/18).

### Data Analysis

All data were analyzed in R Version 3.6 [17].

The *very high risk* group and the *high risk* group were combined for analysis, based on the assumption that this combination included the top 15% of patients with dysphagia risk. The threshold separating the *low risk* group from the combined *high risk* and *very high risk* group was used to calculate measures of discrimination.

Besides using descriptive statistics, the performance of the algorithm was analyzed using measures of discrimination and calibration. As a measure of discrimination, receiver operating-characteristic (ROC) curves with DeLong confidence intervals [18] and AUROC values were used. Confidence intervals (95%) were calculated with 2000 stratified bootstrap replicates using the R pROC package [19]. According to Hosmer et al. [20], an AUROC value above 0.7 is interpreted as acceptable discrimination, a value above 0.8 as excellent, and above 0.9 as outstanding discrimination.

In addition, sensitivity, specificity, positive predictive value (precision), negative predictive value, and accuracy were calculated.

In order to measure calibration, we computed a calibration plot with a 95% confidence interval. The calibration plot illustrates the agreement between the observed frequency of dysphagia patients and the predicted frequency. In the plot, risk predictions are split into deciles and plotted on the *x*-axis, and the corresponding relative frequency of dysphagia is shown on the *y*-axis.

## Results

### Descriptive Statistics

During the evaluation period of more than 12 months, the dysphagia risk was prospectively predicted for 1048 admissions (885 patients) of the internal medicine ward and for 222 admissions (221 patients) of the geriatric ward.

Descriptive statistics for both prospective cohorts are presented in Table 1. Geriatric patients were treated 20 days at median, whereas internal medicine patients were treated 6 days at median. The geriatric department treated more female patients (66.2%) compared to the internal medicine department (46.6%). Regarding the previously coded diagnoses, 2.1% of internal medicine patients had a dysphagia diagnosis coded in a previous hospital admission compared to 0.9% of the geriatric patients. In contrast, 6.8% of the geriatric patients had a coded diagnosis for dementia or Alzheimer in comparison to 5.9% of the internal medicine patients.

### Identification of Dysphagia Patients

For 98 admissions, records of dysphagia or aspiration pneumonia were available in the EHR data of the recent hospital stay. 62 admissions were treated in the internal medicine ward and 36 in the geriatric ward. This resulted in an incidence of dysphagia of 5.9% for internal medicine patients and an incidence of 16.2% for geriatric patients.

Table 3 shows the sources for identification within the EHR system. The majority of internal medicine patients (93.9%) had indications for dysphagia or aspiration pneumonia in the discharge summaries, whereas for the geriatric ward, the majority (80.6%) was coded a relevant procedure, e.g., dysphagia therapy.

**Table 3:** Occurrence of dysphagia and aspiration pneumonia records in different sources within the same EHR system. Multiple sources for patients are possible

	Internal medicine ward	Geriatric ward
ICD-10-GM coded dysphagia (R13)	12 (11.4%)	6 (8.0%)
ICD-10-GM coded aspiration pneumonia (J69)	10 (9.4%)	2 (2.7%)
Discharge summaries	52 (49.5%)	18 (24.0%)
Clinical notes from speech-language pathologists	15 (14.3%)	20 (26.7%)
Procedures for dysphagia	16 (15.2%)	29 (38.7%)

**Fig. 2:**
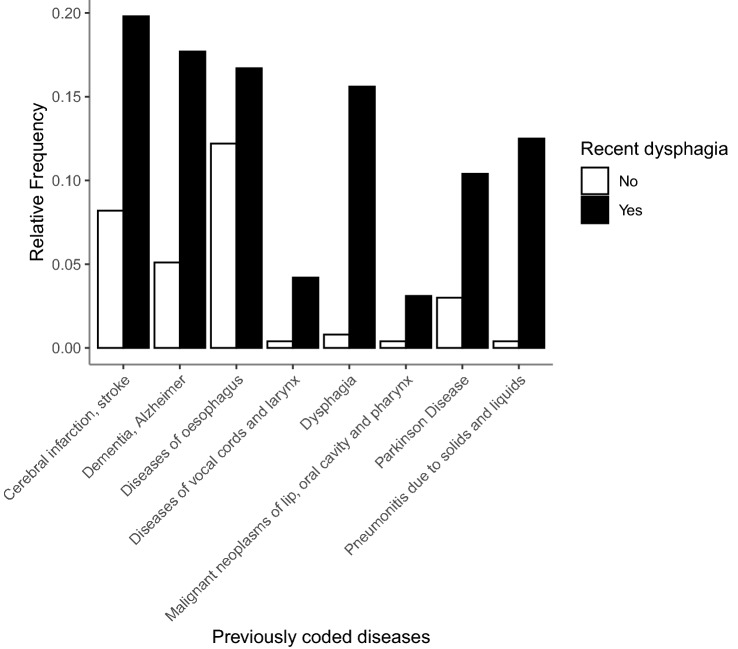
Relative frequencies for previously coded diagnoses in patient history. Results are compared between patients without dysphagia in white and patients with dysphagia in black

In an exploratory analysis, relative frequencies of previous diagnoses were compared between dysphagia and non-dysphagia patients (see Fig. 2). There was a higher relative frequency for dysphagia or aspiration pneumonia (pneumonitis due to solids and liquids) in the patient history of dysphagia patients compared with patients without dysphagia.

### Prospective Performance of the Algorithm

Table 4 presents the results of the prospective prediction for internal medicine patients. For this cohort, the algorithm achieved a specificity of 84.1% [62.3–94.4%], with 829 cases correctly identified as *low risk* cases. The sensitivity in this cohort was 74.2% [62.9–85.5%], with 46 correctly identified dysphagia cases out of a total of 62. Thus, the accuracy for internal medicine patients was 84.5% [83.7–85.2%], the positive predictive value (precision) was 0.227 [0.182–0.246], and the negative predictive value was 0.981 [0.974–0.989].

**Table 4:** Confusion matrix for the prospective prediction of the dysphagia risk prediction tool at the internal medicine ward

		Predicted				Total	
		No dysphagia (low risk)		Dysphagia (high/very high risk)			
		*n*	%	*n*	%	*n*	%
Outcome	No dysphagia	829	**84.1**	157	15.9	986	100.0
	Dysphagia	16	25.8	46	**74.2**	62	100.0
	Total	845	80.6	203	19.4	1048	100.0

Values are presented as absolute frequencies and row percentages

For geriatric patients, results of the prospective predictions are illustrated in Table 5. For this cohort, the algorithm achieved a specificity of 93.0% [68.6–97.7%], with 173 cases correctly identified as *low risk* cases. The sensitivity in this cohort was 44.4% [38.7–58.1%], with 16 correctly identified dysphagia cases out of a total of 36 dysphagia cases. The accuracy for geriatric patients was 85.1% [83.7–85.2%], the positive predictive value (precision) was 0.552 [0.326–0.592], and the negative predictive value was 0.896 [0.868–0.926].

**Table 5:** Confusion matrix for the prospective prediction of the dysphagia risk prediction tool at the geriatric ward

		Predicted				Total	
		No dysphagia (low risk)		Dysphagia (high/very high risk)			
		*n*	%	*n*	%	*n*	%
Outcome	No dysphagia	173	**93.0**	13	7.0	186	100.0
	Dysphagia	20	55.6	16	**44.4**	36	100.0
	Total	193	86.9	29	13.1	222	100.0

Values are presented as absolute frequencies and row percentages

**Fig. 3:**
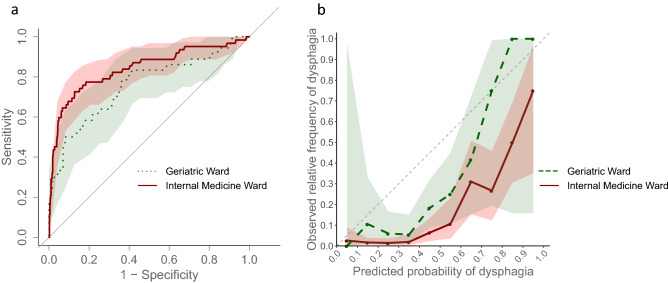
Performance of the algorithm using receiver operating-characteristic (ROC) curves (**a**) and calibration plots (**b**). Prospective predictions for the internal medicine ward are shown in red and for the geriatric ward in green, including 95% confidence intervals

Figure 3a shows the ROC curves for the prospective data for the internal medicine and geriatric ward. The AUROC for the internal medicine ward was 0.841 [0.7781–0.9046] and for the geriatric ward 0.758 [0.6604–0.8550]. Calibration plots for both cohorts are presented in Fig. 3b. For both cohorts, the plots showed a slight overestimation of the dysphagia risk when compared to the observed frequency of the outcome.

## Discussion

In this study, we evaluated a machine learning-based tool predicting the risk of dysphagia in routinely hospitalized patients. The algorithm uses random forest models and EHR data to predict a patient’s risk of dysphagia or aspiration pneumonia. The overall aim was to identify patients with dysphagia as early as possible to prevent aspiration pneumonia and improve care. Like most countries, Austria faces an unprecedented strain on care resources, which is why we believe that prediction models based on ML and AI might provide substantial support in certain health care fields.

During the prospective evaluation in clinical routine of more than twelve months, the algorithm achieved an excellent discrimination for internal medicine patients with an AUROC of 0.84, and only an acceptable discrimination for geriatric patients with an AUROC of 0.76. Several aspects might have led to this performance difference between the two cohorts.

First, compared to general internal medicine patients, patients from geriatric wards have higher risk of dysphagia because of higher age and comorbidities [10, 21]. Therefore, it might be more difficult to distinguish patients with lower risk from those at higher risk within this specific cohort.

Second, although the sensitivity was very low with 44.4% for the risk prediction in geriatric patients, the specificity of the algorithm was high with 93.0%. This provides the possibility to lower the threshold between patients of the *low risk* and *high risk* group, which will shift some patients from *low risk* to *high risk*. Future research needs to determine, whether this could increase the sensitivity and decrease the specificity in order to be more equalized.

Third, the cohort of internal medicine patients was very small with 222 admissions compared to the internal medicine cohort including over 1000 admissions. This also resulted in a big 95%-CI for the AUROC ranging from 0.66 to 0.85. More data are needed to determine the performance of the dysphagia prediction tool in geriatric patients.

Several other models predicting dysphagia or aspiration in dysphagia have been published over the last years. Heijnen et al. [13] developed a logistic regression model to predict aspiration in patients with oropharyngeal dysphagia, but predictors were based on several questionnaires and scales which had to be completed by patients and clinicians. Similarly, Gandolfo et al. [8] developed the predictive dysphagia score to predict persisting dysphagia in stroke patients using body mass index and the modified Ranking Scale. The score, which is based on multivariate logistic regression analysis, predicts dysphagia with a sensitivity of 52.5% [44.5–60.4], a specificity of 89.7% [81.3–95.2], and a fitted AUROC of 0.79 for a sample of 249 patients.

The models by Zhou et al. [6] and Grimm et al. [7] predicted dysphagia in patients with cardiac surgery; while the 38-point RODICS score [7] resulted in an AUROC of 0.75 [0.71–0.80], the bedside scoring model SSG-OD [6] reached an AUROC of 0.83 [0.782–0.884]. However, no results on test data or validation data were reported for both studies.

In comparison to our study, the reviewed models are limited as they can be used only for certain patient populations [6–8], require additional clinical examination [13], or were not tested on separate data sets [6, 7]. Most importantly, the results of our study show the performance of the machine learning-based tool when used in clinical routine instead of test data only.

### Limitations

Due to the COVID-19 pandemic, Austria was in lockdown multiple times since March 2020. Such lockdowns also influence the distributions in EHR data, as mainly severe cases are treated in the hospitals and elective interventions are postponed. During this time, beds at the geriatric wards were occasionally used for patients of other specializations as well. Therefore, the evaluation is slightly impaired due to the pandemic and the unusual situation for hospitals and healthcare. However, this effect is supposed to be higher for the first lockdown in spring 2020 than for the ones within the evaluation period from October 2020 to October 2021.

Another limitation of this study is the use of routinely documented EHR data for outcome classification. Patients with ICD-10-GM coded diagnosis of dysphagia or aspiration pneumonia, dysphagia-related procedures, or evidence in clinical documentation were labeled as dysphagia patients. However, not all cases of dysphagia are detected in clinical routine and hence some patients with dysphagia might have been missed. To overcome this limitation, we aim to validate the prediction of the tool on data assessed by systematic dysphagia screening of speech-language pathologists and specialized physicians in a defined cohort in future.

A further limitation is that this study did not evaluate any preventive measures which were staged for patients in the study cohort. All patients who were predicted a *very high risk* by the dysphagia prediction tool were further examined by speech-language pathologists. Depending on the status of the patient, the examination included a clinical observation, the Gugging Swallowing Screen [22], or, in severer cases, a flexible endoscopic evaluation of swallowing. For patients with identified swallowing disorders, a dietician was consulted and patient care was adapted in order to prevent aspiration pneumonia. Interventions included modification of dietary, feeding assistance, and improvement of the environment for swallowing. Future work should evaluate different processes following the tool’s predictions in order to improve the workflow for preventing aspiration pneumonia.

### Future Research Questions

Some additional aspects need to be addressed in future studies. For training the random forest models in this study, only structured ICD-10-GM coded diagnoses were used as features for prediction. However, clinical notes often contain valuable information in unstructured text format as well. For future developments, such clinical narratives should be pre-processed and included as modeling features for the prediction of dysphagia.

Even though prediction models might be able to discriminate well between dysphagia and non-dysphagia patients, it is essential to determine the clinical usefulness of the models. An important aspect is the acceptance by healthcare professionals and the actual use of such system in clinical routine. A previous evaluation of a similar tool, predicting delirium in hospitalized patients, showed a high user acceptance regarding ease of use and usefulness [23]. Naturally, we aim to include user satisfaction and acceptance of this technology when it comes to predicting dysphagia.

## Conclusion

This study demonstrates the successful implementation and evaluation of a machine learning-based prediction algorithm for dysphagia in clinical routine. During a 13-months evaluation period, the tool predicted the risk of dysphagia for all patients at admission time as well as up to 48 h after admission. Because of the automatic and non-invasive risk prediction without additional effort for healthcare professionals, a broad dysphagia screening was feasible. Although the tool achieved an excellent performance in the internal medicine cohort, more data are needed to determine the performance in geriatric patients, as the sample size in this study was small resulting in big confidence intervals. In future, the use of machine learning-based prediction models might support clinicians in their daily decision-making, leading to fast and personalized preventive actions and thus increasing patient care.

## Supplementary Information

Below is the link to the electronic supplementary material.

Supplementary file1 (DOCX 483 KB)

Supplementary file2 (XLSX 64 KB)
