# Supplementary material for: Evaluation of a Machine Learning-Based Dysphagia Prediction Tool in Clinical Routine: A Prospective Observational Cohort Study
Source: Dysphagia. 2023 Jan 10;38(4):1238–46. doi: 10.1007/s00455-022-10548-9 (PMC9831015; doi:10.1007/s00455-022-10548-9)
Supplement: Supplementary file 1 — Supplementary file1 (DOCX 483 KB) [file 455_2022_10548_MOESM1_ESM.docx]

**Supplement A**

**Table A1.** Discriminative performance of the three random forest models integrated in the tool for prediction at admission time (Model A), recalculation at evening of admission (Model B) and recalculation at the second evening of admission (Model C).

| **Model** | **Prediction time** | **AUROC** | **95%-CI** |
| --- | --- | --- | --- |
| A | Admission | 0.898 | 0.889 - 0.906 |
| B | Evening 1 | 0.928 | 0.921 - 0.935 |
| C | Evening 2 | 0.938 | 0.931 - 0.944 |

**Table A2.** German search terms for the identification of patients with dysphagia or aspiration pneumonia when using clinical notes and discharge summaries. Approximate string match was used with different distances in order to account for typing errors.

| **German (original search term)** | **English (translated version)** |
| --- | --- |
| Aspiration, Aspirier* | aspiration |
| Drooling | drooling |
| Dysphagie, Schluckstörung | dysphagia, |
| Dysphagietherapie, FDT, Funktionelle Dysphagietherapie | (functional) dysphagia therapy |
| FEES, Flexible endoskopische Evaluation des Schluckens | fiberoptic endoscopic evaluation of swallowing |
| GUSS | Gugging Swallowing Screen |
| Hinweise auf pharyngeale Retentionen | Evidence of pharyngeal retention |
| Husten direkt nach dem Trinken, Husten direkt nach der Nahrungsaufnahme | Cough immediately after drinking/after eating |
| Jejunalsonde | jejunal tube |
| klinische Schluckuntersuchung, klinischer Schluckversuch, KSU, Schluckuntersuchung | clinical swallowing examination |
| nasobiliäre Sonde | nasobiliary tube |
| Orales Absaugen | oral suctioning |
| PEG, PEG-Sonde | percutaneous endoscopic gastrostomy (PEG) tube |
| Presbyphagie | presbyphagia |
| sammelt in der Wangentasche | remnants of food remaining in the cheek pouches |
| Schluckberatung | Swallowing advice |
| unkoordiniertes Abschlucken | uncoordinated swallowing |
| verlängerte orale Phase, verlängerte orale Vorbereitungsphase | prolonged oral transit time |
| verzögerter Hustenreflex, verzögerter Hustenreiz | Delayed cough reflex |
| VFS, VFSS, Videofluoroskopie, Videofluoroskopische Evalutation des Schluckens, Videofluoroskopische Schluckstudie, Videofluoroskopische Schluckuntersuchung | videofluoroscopic swallowing study (VFSS) |
| wet voice, wetvoice | wet voice |

**
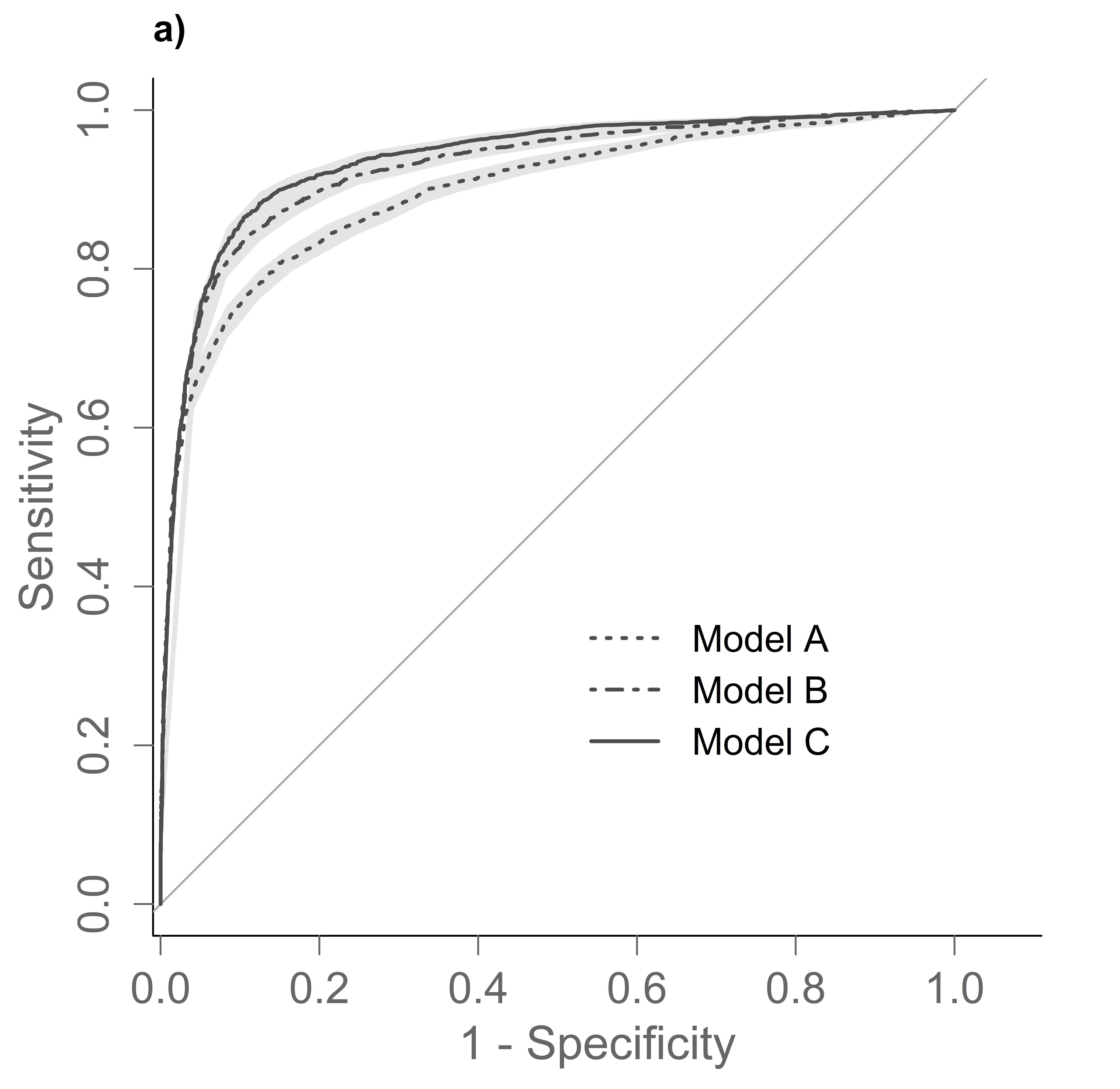
**


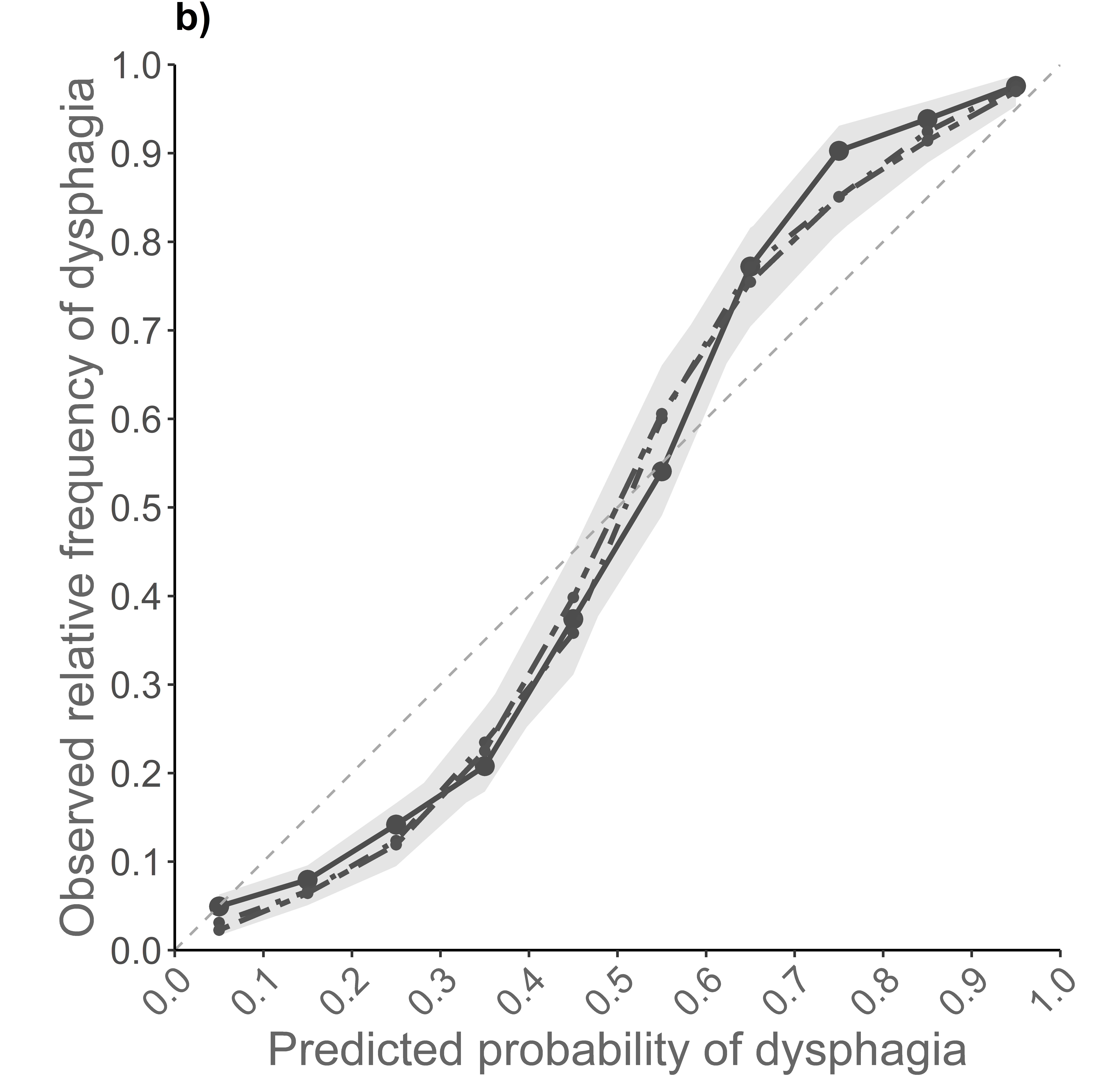


**Fig. A1**. Performance of the algorithm using Receiver Operating Characteristic (ROC) curves (a) and calibration plots (b) of the three models A, B, C integrated in the prediction tool, including 95% confidence intervals. Corresponding AUROC values are shown in Table A1.
